# A Novel Automated Approach for Infrared-Based Assessment of Meibomian Gland Morphology

**DOI:** 10.1167/tvst.8.4.17

**Published:** 2019-08-02

**Authors:** Clara Llorens-Quintana, Laura Rico-del-Viejo, Piotr Syga, David Madrid-Costa, D. Robert Iskander

**Affiliations:** 1 Wroclaw University of Science and Technology, Faculty of Fundamental Problems of Technology, Department of Biomedical Engineering, Wroclaw, Poland; 2 Complutense University of Madrid, Faculty of Optics and Optometry, Department of Optometry and Vision, Madrid, Spain; 3 Wroclaw University of Science and Technology, Faculty of Fundamental Problems of Technology, Department of Computer Science, Wroclaw, Poland

**Keywords:** meibomian glands, infrared meibography, image processing, objective medical image analysis

## Abstract

**Purpose:**

We present and validate a new methodology for analyzing, in an automated and objective fashion, infrared images of the meibomian glands (MG).

**Methods:**

The developed algorithm consists of three main steps: selection of the region of interest, detection of MG, and analysis of MG morphometric parameters and dropout area (DOA). Additionally, a new approach to quantify the irregularity of MG is introduced. We recruited 149 adults from a general population. Infrared meibography, using Keratograph 5M, was performed. Images were assessed and graded subjectively (Meiboscore) by two experienced clinicians and objectively with the proposed automated method.

**Results:**

The correlation of subjective DOA assessment between the two clinicians was poor and the average percentage of DOA estimated objectively for each Meiboscore group did not lie within their limits. The objective assessment showed lower variability of meibography grading than that obtained subjectively. Additionally, a new grading scale of MG DOA that reduces intraclass variation is proposed. Reported values of MG length and width were inversely proportional to the DOA. Gland irregularity was objectively quantified.

**Conclusions:**

The proposed automatic and objective method provides accurate estimates of the DOA as well as additional morphologic parameters that could add valuable information in MG dysfunction understanding and diagnosis.

**Translational Relevance:**

This approach highlights the shortcomings of currently used subjective methods, and provides the clinicians with an objective, quantitative and less variable alternative for assessing MG in a noninvasive and automated fashion. It provides a viable alternative to more time-consuming subjective methods.

## Introduction

Meibomian glands (MG) are large sebaceous glands placed on the tarsal conjunctiva of the eye in parallel arrangement. They are responsible for synthetizing and secreting lipids (known as meibum) onto the eye surface, coating the aqueous layer of the tear film.^1^ Meibum is responsible for reducing tear film evaporation, enhancing its stability and spreading, as well as preventing the contamination of the tear film by sebum.^2^,^3^ Therefore, tear physiology strongly depends on properly functioning MG.^4^ A functional or structural problem of these glands can cause meibomian gland dysfunction (MGD).^5^,^6^ During the last decades, significant attention has been paid to MGD as it has been found to be a major cause of evaporative dry eye,^7^–^10^ a disease affecting the quality of life of those who suffer it.^11^ One of the clinical observable signs in MGD is the atrophy of the glands that leads to loss of glandular tissue (i.e., MG drop out).^12^ By directly observing the morphology of MG, their structure and the drop out can be assessed.^13^

Meibography is an optical imaging technique used for in vivo visualization of MG morphology allowing the estimation of the dropout area (DOA).^14^,^15^ There are two different approaches to meibography, both of them use infrared illumination and require eversion of the eyelid. One method is performed using transillumination of the eyelid^16^,^17^ and the other, known as noncontact meibography, uses direct illumination.^15^,^18^,^19^ The latter has become more popular as is less uncomfortable for the patient, can image larger areas, and is easier to perform for the practitioner.^18^ Nowadays, commercially available instruments incorporate an option to acquire MG infrared images.

To classify the acquired meibography images, different grading scales have been proposed, in which the images are categorized according to the estimated DOA.^14^,^18^,^20^,^21^ Nonetheless, these classifications and assessment of the images are performed subjectively and manually, or in the best case, semiautomatically. Subjective grading is very inconsistent; consequently there is a decrease in measurement repeatability and agreement between raters and also an increase in the diagnostic time.^20^ Objective grading of the DOA could result in a better intra- and interrater measurement repeatability and agreement.^14^,^22^–^24^

**Figure 1:**
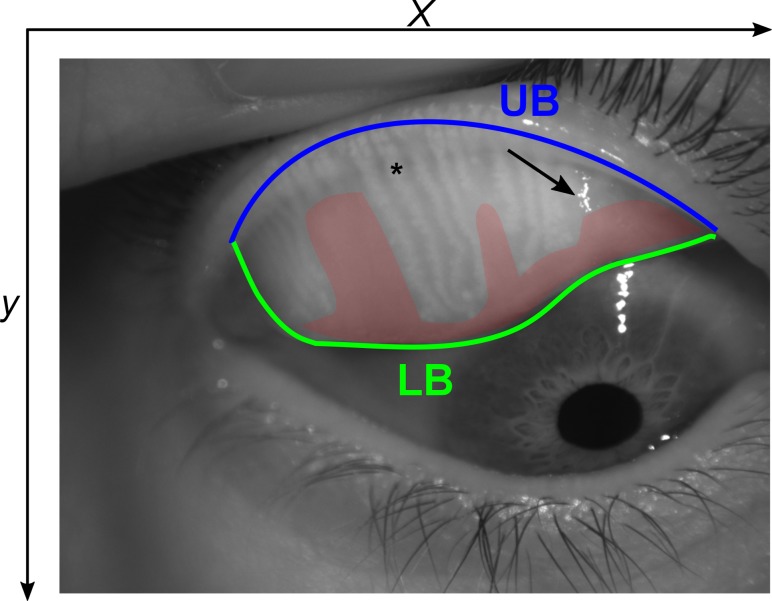
An example of an infrared meibography image with the upper (UB) and lower (LB) boundaries of the ROI outlined in blue and green lines, respectively. The red shaded area is the estimated dropout area, the arrow indicates specular reflection, and the asterisks denotes a single Meibomian gland.

Developing an automated method to objectively analyze the meibography images that could work in the majority of the cases is challenging, since those images often have low contrast, nonuniform illumination, specular reflections, defocused areas, and presence of artifacts. In addition, pixel intensity changes gradually between the background and glands making glands segmentation more difficult. Also, the position of the tarsal conjunctiva may be different in each meibography acquisition. When assessing the images, it is important to differentiate pixels into three categories, that is those that belong to a gland, an intergland region (i.e., healthy tissue with no glands), and areas of tissue loss (see Fig. 1).

To date, only few fully automated algorithms for analyzing meibography images have been proposed. They classify meibography images according to the area of MG loss^25^–^27^ or according to MG morphology.^28^ Analysis of morphometric parameters of MG, such as gland length, width, and regularity, may provide additional value in MGD diagnosis.^14^,^23^,^24^ An objective and quantitative method would allow assessing subtle changes of MG in patient monitoring.^29^

We developed and validated a versatile and robust automated algorithm for analyzing the MG DOA as well as the morphometric parameters of the glands of the upper eyelid to provide new features for MG assessment and classification.

## Methods

### Clinical Evaluation

Clinical examinations were performed at the Optometry Clinic of the Complutense University of Madrid. A total of 149 adult volunteers (85 males and 74 females, mean age ± standard deviation = 42 ± 17 years; range, 18–88 years) were recruited for the study. This general population cohort has varying levels of MG health. The study was approved by the ethics committee (CEIC) of the Hospital Clínico San Carlos of Madrid and adhered to the tenets of the Declaration of Helsinki. All participants signed a written informed consent after explanation of the purpose and possible consequences of the study. Exclusion criteria included contact lens wear in the last 24 hours before the exam, ocular complications within the three months before the exam, eyelid margin abnormalities or difficulties to perform the eversion of the eyelid.

Meibography of the upper eyelid was performed by an experienced clinician only in the right eye of each patient. Two masked clinicians subjectively graded all acquired images using the Meiboscore grading scale proposed by Arita et al.,^18^ where each subject is classified in one of four groups according to the total percentage of DOA as follows: (1) Meiboscore 0: DOA = 0, (2) Meiboscore 1: 0% < DOA ≤ 32%, (3) Meiboscore 2: 32% < DOA ≤ 65% and (4) Meiboscore 3: DOA > 65%.

Later, the images also were objectively analyzed with the proposed algorithm and the area of dropout was objectively determined to classify the subjects into the aforementioned groups. Additionally, the morphologic parameters objectively extracted were compared along the different grades.

### Equipment

MG images were acquired using Keratograph 5M (Oculus Optikgerate, Germany). This instrument acquires RGB infrared images with a resolution of 1360 × 1024 px. Raw images were exported and stored as bitmaps to analyze them offline. The developed algorithm was divided into three steps: (1) selection of the region of interest (ROI), which is the tarsal conjunctiva; (2) detection of each gland; and (3) examination, divided into the global analysis of the DOA and the local analysis of the morphologic characteristics of the glands. These steps are elaborated in detail in the next three subsections.

### ROI Selection

**Figure 2:**
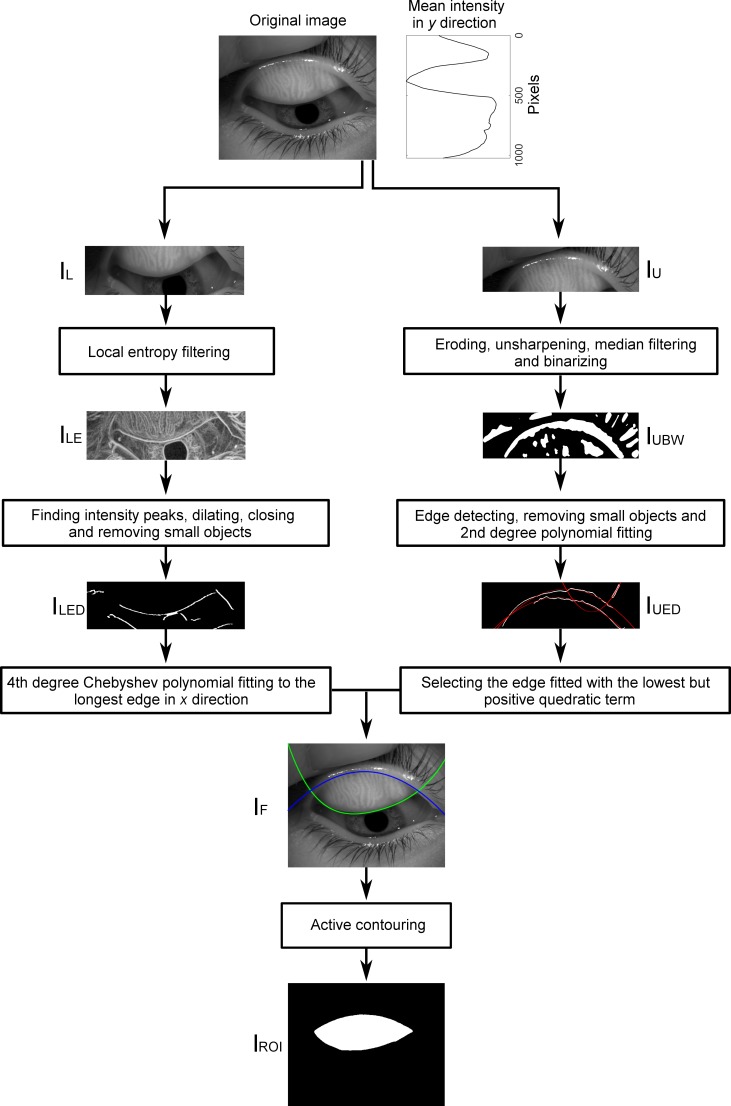
The algorithm for estimating the ROI. For details, see text.

Figure 1 shows an example of an acquired image where the ROI, consisting of the glands and DOA, is demarcated. Automatic selection of the ROI was performed by identifying separately the upper and lower boundary of the everted eyelid. For this, the original image was converted to grayscale and split in two, which was done by exploring the mean intensity levels of the grayscale image along the *y* direction (Figs. 2I_U_, 2I_L_).

For obtaining the upper boundary, image I_U_ was eroded using a disk-shaped structural element with a radius of 10 px; the size of the structural element was chosen so that high spatial frequencies were removed whereas objects with low spatial frequencies were preserved. The upper boundary is a large and convex structure. To detect the wide objects, the upper image was then sharpened using a Gaussian low pass filter with a standard deviation of 30 px and filtered in a 40 × 40 px neighborhood with a median filter to reduce small irregularities and soften the edges. The resulting image was then binarized (Fig. 2I_UBW_) and the edges of the remaining objects were detected using Sobel filter with horizontal orientation. To remove those small resulting edges that may not belong to the upper boundary of the eyelid, all objects with less than, an empirically found, threshold of 500 px were removed. All remaining edges were fitted with a second degree polynomial in *x* (Fig. 2I_UED_), which adequately described the main shape of the upper boundary. Due to the morphologic characteristics of the everted eyelid, the polynomial with the smallest positive coefficient in the quadratic term corresponded to the upper boundary of the ROI (Fig. 2I_F_).

The lower boundary of the ROI is a high-gradient edge which is identified by applying a local entropy filter to image I_L_ in a neighborhood of 9 × 9 px (Fig. 2I_LE_).^30^ This neighborhood size was determined empirically so that the small local irregularities did not have enough weight while the information on the edge was preserved. Then, the intensity peaks of the filtered image were identified and a binary image with their position was created. Pixels belonging to the same structure were joined by dilating the image with a horizontal rectangle-shaped structural element of size 3 × 12 px and closing with a disk-shaped structural element with a radius of 6 px. Objects with less than 500 px were removed as they may belong to other edge of the lower boundary of the eyelid as those from the eye lashes or pupil (Fig. 2I_LED_).

From the remaining objects, the lower boundary of the eyelid corresponded to the largest object along the *x* direction. The boundary of this object was fitted with a fourth degree Chebyshev polynomial of the first kind in *x*.^31^ Chebyshev polynomials were chosen due to their property of orthogonality that allows constraining the fourth term to be negative according to the concave shape of the lower boundary of the everted eyelid (Fig. 2I_F_). The initial estimate of the ROI was the area defined by the fitted functions to the upper and lower boundaries of the everted eyelid, which was used as an initial mask for performing active contouring using the Chan-Vese method on the original image filtered with a local standard deviation filter in a neighborhood of 9 × 9 px.^32^ The resulting binary image was the final ROI where the detection of the gland would be performed (Fig. 2I_ROI_).

### Glands Segmentation

**Figure 3:**
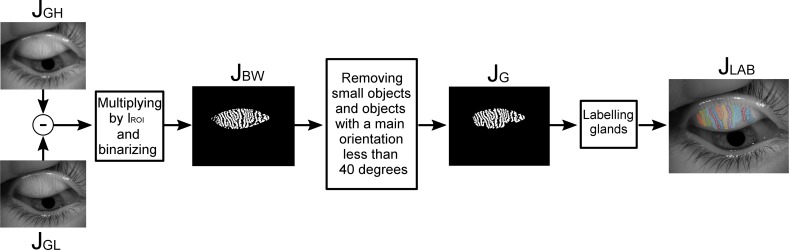
The main steps of the gland segmentation algorithm consisting of filtering, morphologic, and labeling operations. For details, see text.

To isolate the glands, the original gray scale image was convoluted in parallel with two different Gaussians kernels; one having high standard deviation of 30 px (Fig. 3J_GH_), which suppresses high-frequency spatial information and the other with low standard deviation of 2 px (Fig. 3J_GL_). This band-pass filtering increased the visibility of the MG edges without enhancing random noise. The resulting image then was multiplied by the binary mask of the ROI. This image had very low contrast so it was binarized using an adaptive threshold with a high sensitivity value (i.e., thresholding more pixels as foreground) and then the shape of the gland was smoothed by applying a median filter in a rectangular neighborhood of size 5 × 3 px (Fig. 3J_BW_), determined empirically. To remove the remaining noise from the background, all objects containing less than 800 px were removed. It must be considered that the ROI tends to overestimate the tarsal conjunctiva area so as not to lose information of the glands. As a result, it could have happened that some detected objects did not belong to a gland, but to an edge of the eyelid. Given that glands have a mostly vertical orientation while eyelid edges are horizontal, all objects with a main orientation, estimated as the angle of the best-fit ellipse to the given object, lower than 40° or higher than 140° were removed (both angles conservatively determined empirically), resulting in the final binary image of the glands (Fig. 3J_G_).

**Figure 4:**
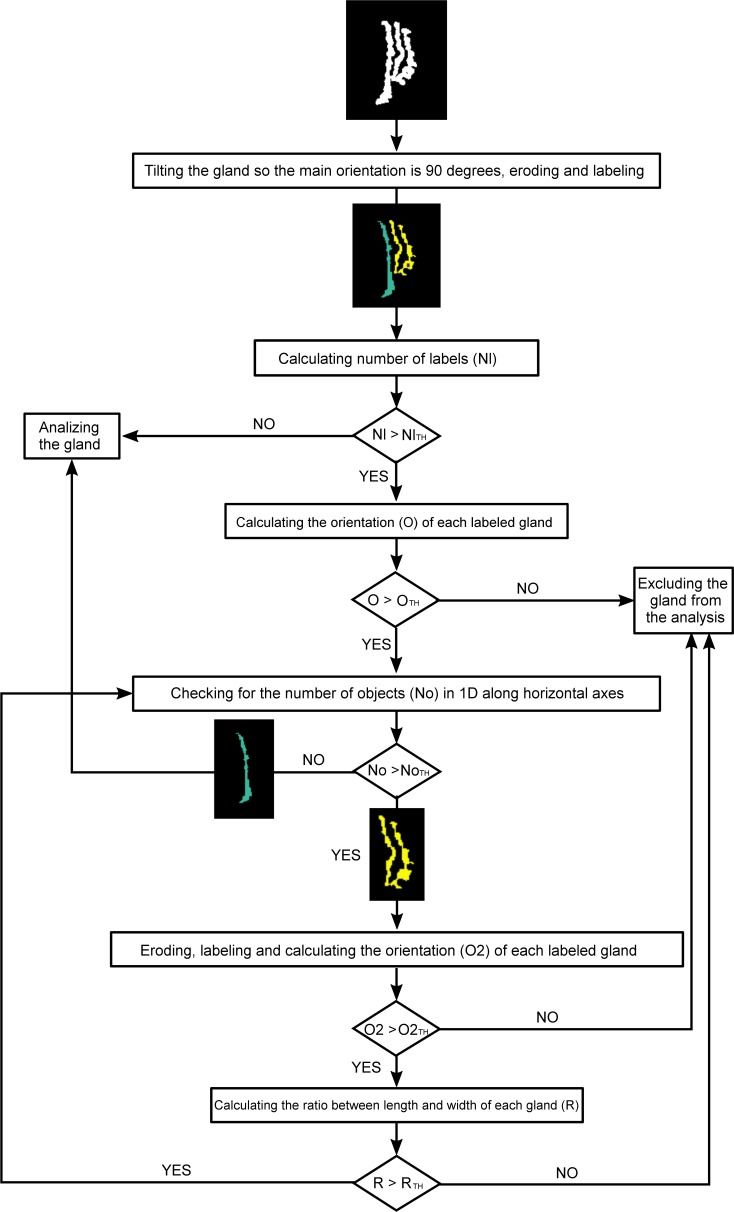
Algorithm used to fragment glands that have been labeled together as a single gland. The input is an example of three grands that have been labeled as a single gland. For images acquired with the Keratograph 5M, the threshold values are: NlTH = 1, OTH = 50°, NoTH = 1, O2TH = 50°, and RTH = 3.

To perform local analysis of the morphologic characteristics of the glands, each gland must be detected and analyzed separately. Thus, the detected glands were labeled and studied individually (Fig. 3J_LAB_). In some cases, the glands appeared to be connected in a fork-like manner, resulting in a single label for more than one gland. This was overcome by applying the fragmentation algorithm depicted in a chart shown in Figure 4.

### MG Analyses

#### Automatic DOA Estimation

To estimate the DOA, it is important to differentiate between the intergland regions and the DOA. These regions are identical in appearance and both are classified as background, but the first belongs to healthy tissue whereas the second to the DOA. Anatomically, the width of intergland regions is smaller than the width of the glands,^33^ which is of the order of 20 px for images acquired with Keratograph 5M. Hence, any area classified as background wider than 20 px should belong to a DOA and not to an intergland area. Accordingly, having the knowledge on the mean gland width, the binary image containing the detected glands (Fig. 3J_G_) was filtered with a 2D rotational disk filter of 20 px radius. Due to the high reflective properties of the tarsal conjunctiva, regions with specular reflections inside the ROI may appear. These regions were identified on the original gray scale image as pixels with intensity values greater than 200 and they were not taken into account in the DOA calculation.

The percentage of the DOA is given by:

$$\newcommand{\bialpha}{\boldsymbol{\alpha}}\newcommand{\bibeta}{\boldsymbol{\beta}}\newcommand{\bigamma}{\boldsymbol{\gamma}}\newcommand{\bidelta}{\boldsymbol{\delta}}\newcommand{\bivarepsilon}{\boldsymbol{\varepsilon}}\newcommand{\bizeta}{\boldsymbol{\zeta}}\newcommand{\bieta}{\boldsymbol{\eta}}\newcommand{\bitheta}{\boldsymbol{\theta}}\newcommand{\biiota}{\boldsymbol{\iota}}\newcommand{\bikappa}{\boldsymbol{\kappa}}\newcommand{\bilambda}{\boldsymbol{\lambda}}\newcommand{\bimu}{\boldsymbol{\mu}}\newcommand{\binu}{\boldsymbol{\nu}}\newcommand{\bixi}{\boldsymbol{\xi}}\newcommand{\biomicron}{\boldsymbol{\micron}}\newcommand{\bipi}{\boldsymbol{\pi}}\newcommand{\birho}{\boldsymbol{\rho}}\newcommand{\bisigma}{\boldsymbol{\sigma}}\newcommand{\bitau}{\boldsymbol{\tau}}\newcommand{\biupsilon}{\boldsymbol{\upsilon}}\newcommand{\biphi}{\boldsymbol{\phi}}\newcommand{\bichi}{\boldsymbol{\chi}}\newcommand{\bipsi}{\boldsymbol{\psi}}\newcommand{\biomega}{\boldsymbol{\omega}}{\rm{DO}}{{\rm{A}}} = {{{N_{{\rm{px}}}}_{{\rm{ROI}}} - {N_{{\rm{pxGL}}}} - {N_{{\rm{pxR}}}}} \over {{N_{{\rm{pxROI}}}}}} \times 100,$$

where $\newcommand{\bialpha}{\boldsymbol{\alpha}}\newcommand{\bibeta}{\boldsymbol{\beta}}\newcommand{\bigamma}{\boldsymbol{\gamma}}\newcommand{\bidelta}{\boldsymbol{\delta}}\newcommand{\bivarepsilon}{\boldsymbol{\varepsilon}}\newcommand{\bizeta}{\boldsymbol{\zeta}}\newcommand{\bieta}{\boldsymbol{\eta}}\newcommand{\bitheta}{\boldsymbol{\theta}}\newcommand{\biiota}{\boldsymbol{\iota}}\newcommand{\bikappa}{\boldsymbol{\kappa}}\newcommand{\bilambda}{\boldsymbol{\lambda}}\newcommand{\bimu}{\boldsymbol{\mu}}\newcommand{\binu}{\boldsymbol{\nu}}\newcommand{\bixi}{\boldsymbol{\xi}}\newcommand{\biomicron}{\boldsymbol{\micron}}\newcommand{\bipi}{\boldsymbol{\pi}}\newcommand{\birho}{\boldsymbol{\rho}}\newcommand{\bisigma}{\boldsymbol{\sigma}}\newcommand{\bitau}{\boldsymbol{\tau}}\newcommand{\biupsilon}{\boldsymbol{\upsilon}}\newcommand{\biphi}{\boldsymbol{\phi}}\newcommand{\bichi}{\boldsymbol{\chi}}\newcommand{\bipsi}{\boldsymbol{\psi}}\newcommand{\biomega}{\boldsymbol{\omega}}{N_{{\rm{pxROI}}}}$ is the number of pixels occupied by the ROI, $\newcommand{\bialpha}{\boldsymbol{\alpha}}\newcommand{\bibeta}{\boldsymbol{\beta}}\newcommand{\bigamma}{\boldsymbol{\gamma}}\newcommand{\bidelta}{\boldsymbol{\delta}}\newcommand{\bivarepsilon}{\boldsymbol{\varepsilon}}\newcommand{\bizeta}{\boldsymbol{\zeta}}\newcommand{\bieta}{\boldsymbol{\eta}}\newcommand{\bitheta}{\boldsymbol{\theta}}\newcommand{\biiota}{\boldsymbol{\iota}}\newcommand{\bikappa}{\boldsymbol{\kappa}}\newcommand{\bilambda}{\boldsymbol{\lambda}}\newcommand{\bimu}{\boldsymbol{\mu}}\newcommand{\binu}{\boldsymbol{\nu}}\newcommand{\bixi}{\boldsymbol{\xi}}\newcommand{\biomicron}{\boldsymbol{\micron}}\newcommand{\bipi}{\boldsymbol{\pi}}\newcommand{\birho}{\boldsymbol{\rho}}\newcommand{\bisigma}{\boldsymbol{\sigma}}\newcommand{\bitau}{\boldsymbol{\tau}}\newcommand{\biupsilon}{\boldsymbol{\upsilon}}\newcommand{\biphi}{\boldsymbol{\phi}}\newcommand{\bichi}{\boldsymbol{\chi}}\newcommand{\bipsi}{\boldsymbol{\psi}}\newcommand{\biomega}{\boldsymbol{\omega}}{N_{{\rm{pxGL}}}}$ the number of pixels occupied by the blurred image of the glands (containing glands and intergland area) and $\newcommand{\bialpha}{\boldsymbol{\alpha}}\newcommand{\bibeta}{\boldsymbol{\beta}}\newcommand{\bigamma}{\boldsymbol{\gamma}}\newcommand{\bidelta}{\boldsymbol{\delta}}\newcommand{\bivarepsilon}{\boldsymbol{\varepsilon}}\newcommand{\bizeta}{\boldsymbol{\zeta}}\newcommand{\bieta}{\boldsymbol{\eta}}\newcommand{\bitheta}{\boldsymbol{\theta}}\newcommand{\biiota}{\boldsymbol{\iota}}\newcommand{\bikappa}{\boldsymbol{\kappa}}\newcommand{\bilambda}{\boldsymbol{\lambda}}\newcommand{\bimu}{\boldsymbol{\mu}}\newcommand{\binu}{\boldsymbol{\nu}}\newcommand{\bixi}{\boldsymbol{\xi}}\newcommand{\biomicron}{\boldsymbol{\micron}}\newcommand{\bipi}{\boldsymbol{\pi}}\newcommand{\birho}{\boldsymbol{\rho}}\newcommand{\bisigma}{\boldsymbol{\sigma}}\newcommand{\bitau}{\boldsymbol{\tau}}\newcommand{\biupsilon}{\boldsymbol{\upsilon}}\newcommand{\biphi}{\boldsymbol{\phi}}\newcommand{\bichi}{\boldsymbol{\chi}}\newcommand{\bipsi}{\boldsymbol{\psi}}\newcommand{\biomega}{\boldsymbol{\omega}}{N_{{\rm{pxR}}}}$ the number of pixels belonging to a specular reflection.

#### Morphometric Parameters of the Glands

To better characterize the glands, three main morphometric parameters were obtained: gland length, gland width, and gland irregularity. To perform this analysis, each detected gland was evaluated separately. However, to have a general measure for all glands in the tarsal conjunctiva that could describe the overall condition of the MG, mean values of length, width, and irregularity were computed for each eyelid.

For the individual gland analysis an ellipse with the same normalized second central moment as the gland was fitted and the gland was rotated so its main orientation was vertical. The major and minor axes length of this ellipse were considered as the approximated length and width of the gland, respectively. Absolute value of MG length depends on how the eyelid is everted, so if a small amount of eyelid is everted we could have ended up with an unrealistically short MG length. To overcome this, the relative length of the gland with respect to the height of the everted eyelid also was computed.

**Figure 5:**
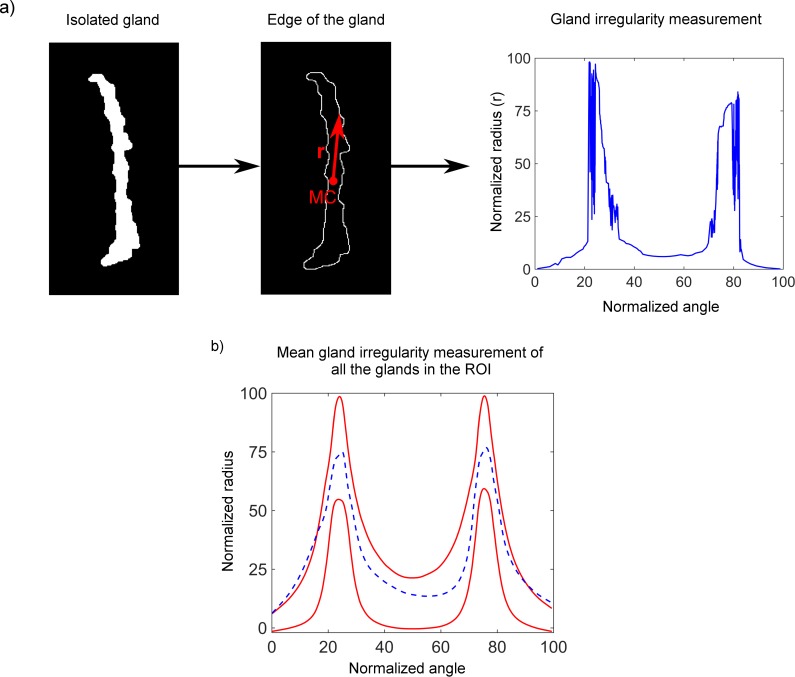
(a) Proposed irregularity measurement of a single gland. MC is the position of the mass center of the gland and r the radial coordinates from MC to the edge that are computed for 360°. Normalized values of the radius are represented in a polar plot (Fig. 5a, right). (b) Representation of the gland irregularity for all glands in the ROI. Red lines represent mean ± 1 SD of the radial coordinates of 200 regular glands. Blue dashed line represents the mean radial coordinates of all the glands for a given eye. The more the blue dashed line protrudes the red lines, the greater the irregularity of the glands is in the given eye.

To study the shape of the gland, the boundary of the binary image of the gland was traced. A healthy gland is elongated and thin.^33^ Hence, in general terms, the irregularity of the gland is defined as the difference of its shape from the shape of a regular gland. Further, the gland shape is described by locating the mass center of the gland, which is used as the origin to convert the boundary coordinates from Cartesian to polar (see Fig. 5a). Since we were interested in the shape rather than the size, for the irregularity measurement the radial values were normalized avoiding length and width influence. To quantify the amount of irregularity of all MG, the polar coordinates of the boundaries of 200 regular glands from the upper eyelid have been computed and the mean ± 1 SD was considered as the limit for a regular gland. The 200 regular glands were taken from meibography images acquired in this study. These limits were represented in a normalized one-dimensional (1D) radial plot (red lines in Fig. 5b) together with the mean radial value of all glands in the tarsal conjunctiva for the given eye (blue dashed line in Fig. 5b). The area enclosed between the red and blue lines out of the limits was computed using trapezoidal numerical integration. This area quantified the irregularity of the gland.

## Results

**Figure 6:**
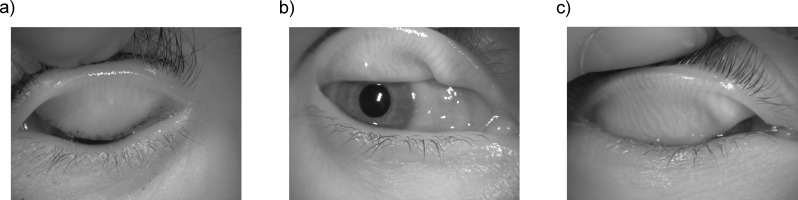
Example of three images where the algorithm was unable to detect the correct ROI due to a problem during the acquisition. (a) Unfocused image (focus is at the eyelashes), (b) out of frame image, and (c) image where the upper eyelid is attached to the lower eyelid.

Automated and objective analysis of the glands was performed in a batch mode with no user input. Manual adjustment consisting of ROI selection was necessary in nine images (6.04%). In six of those cases, the images were not acquired properly, while in the remaining three images, a preprocessing problem was encountered. Acquisition problem can be due to an unfocused image, off center image with part of the tarsal conjunctiva out of the frame, or because the lower boundary of the upper eyelid is attached to the lower eyelid (see Fig. 6). It is noteworthy that the algorithm never failed in the steps after the ROI selection.

The set of subjects was divided into 58 subjects with Meiboscore 0, 63 with Meiboscore 1, 22 with Meiboscore 2, and six with Meiboscore 3, according to grader 1 subjective criteria. For this distribution of subjects, the mean ± 1 SD values of the objectively estimated percentage of DOA were 12.55 ± 9.89, 20.71 ± 9.96, 29.17 ± 13.45, and 54.83 ± 12.30, respectively for Meiboscores 0, 1, 2, and 3. According to grader 2 subjective criteria, there were 55 subjects with Meiboscore 0, 66 with Meiboscore 1, 19 with Meiboscore 2, and nine with Meiboscore 3. For this distribution of subjects, the mean ± 1 SD values of the objectively estimated percentage of DOA were 12.04 ± 8.84, 19.56 ± 9.31, 30.63 ± 12.24, and 53.00 ± 10.19, respectively for Meiboscores 0, 1, 2, and 3.

Despite having only four choices, the intergrader variability was high for the subjective Meiboscore resulting in Spearman's correlation coefficient of $\newcommand{\bialpha}{\boldsymbol{\alpha}}\newcommand{\bibeta}{\boldsymbol{\beta}}\newcommand{\bigamma}{\boldsymbol{\gamma}}\newcommand{\bidelta}{\boldsymbol{\delta}}\newcommand{\bivarepsilon}{\boldsymbol{\varepsilon}}\newcommand{\bizeta}{\boldsymbol{\zeta}}\newcommand{\bieta}{\boldsymbol{\eta}}\newcommand{\bitheta}{\boldsymbol{\theta}}\newcommand{\biiota}{\boldsymbol{\iota}}\newcommand{\bikappa}{\boldsymbol{\kappa}}\newcommand{\bilambda}{\boldsymbol{\lambda}}\newcommand{\bimu}{\boldsymbol{\mu}}\newcommand{\binu}{\boldsymbol{\nu}}\newcommand{\bixi}{\boldsymbol{\xi}}\newcommand{\biomicron}{\boldsymbol{\micron}}\newcommand{\bipi}{\boldsymbol{\pi}}\newcommand{\birho}{\boldsymbol{\rho}}\newcommand{\bisigma}{\boldsymbol{\sigma}}\newcommand{\bitau}{\boldsymbol{\tau}}\newcommand{\biupsilon}{\boldsymbol{\upsilon}}\newcommand{\biphi}{\boldsymbol{\phi}}\newcommand{\bichi}{\boldsymbol{\chi}}\newcommand{\bipsi}{\boldsymbol{\psi}}\newcommand{\biomega}{\boldsymbol{\omega}}{r^2} = 0.50$. Both graders coincided in Meiboscore grade in 65% of the cases. To assess the agreement between both graders, the κ statistic and its statistical significance were computed. The κ statistic, proposed by Landis and Koch,^34^ is used to assess the agreement when the measuring scale is ordinal—it indicates the proportion of agreement taking into account the expected agreement by chance. The agreement between graders was moderate $\newcommand{\bialpha}{\boldsymbol{\alpha}}\newcommand{\bibeta}{\boldsymbol{\beta}}\newcommand{\bigamma}{\boldsymbol{\gamma}}\newcommand{\bidelta}{\boldsymbol{\delta}}\newcommand{\bivarepsilon}{\boldsymbol{\varepsilon}}\newcommand{\bizeta}{\boldsymbol{\zeta}}\newcommand{\bieta}{\boldsymbol{\eta}}\newcommand{\bitheta}{\boldsymbol{\theta}}\newcommand{\biiota}{\boldsymbol{\iota}}\newcommand{\bikappa}{\boldsymbol{\kappa}}\newcommand{\bilambda}{\boldsymbol{\lambda}}\newcommand{\bimu}{\boldsymbol{\mu}}\newcommand{\binu}{\boldsymbol{\nu}}\newcommand{\bixi}{\boldsymbol{\xi}}\newcommand{\biomicron}{\boldsymbol{\micron}}\newcommand{\bipi}{\boldsymbol{\pi}}\newcommand{\birho}{\boldsymbol{\rho}}\newcommand{\bisigma}{\boldsymbol{\sigma}}\newcommand{\bitau}{\boldsymbol{\tau}}\newcommand{\biupsilon}{\boldsymbol{\upsilon}}\newcommand{\biphi}{\boldsymbol{\phi}}\newcommand{\bichi}{\boldsymbol{\chi}}\newcommand{\bipsi}{\boldsymbol{\psi}}\newcommand{\biomega}{\boldsymbol{\omega}}(\kappa = 0.463,{\rm{\ }}P \lt 0.001)$.

It can be seen that the objectively assessed percentage of the DOA does not correspond to the limits established for Meiboscore and that it is associated with high standard deviation. When applying the Meiboscore percentage limits (i.e., 0, 1–32, 33–65, and 66–100) to the objectively estimated DOA calculated with the proposed automated algorithm, the correlation between the subjective and objective classification is poor (Spearman's $\newcommand{\bialpha}{\boldsymbol{\alpha}}\newcommand{\bibeta}{\boldsymbol{\beta}}\newcommand{\bigamma}{\boldsymbol{\gamma}}\newcommand{\bidelta}{\boldsymbol{\delta}}\newcommand{\bivarepsilon}{\boldsymbol{\varepsilon}}\newcommand{\bizeta}{\boldsymbol{\zeta}}\newcommand{\bieta}{\boldsymbol{\eta}}\newcommand{\bitheta}{\boldsymbol{\theta}}\newcommand{\biiota}{\boldsymbol{\iota}}\newcommand{\bikappa}{\boldsymbol{\kappa}}\newcommand{\bilambda}{\boldsymbol{\lambda}}\newcommand{\bimu}{\boldsymbol{\mu}}\newcommand{\binu}{\boldsymbol{\nu}}\newcommand{\bixi}{\boldsymbol{\xi}}\newcommand{\biomicron}{\boldsymbol{\micron}}\newcommand{\bipi}{\boldsymbol{\pi}}\newcommand{\birho}{\boldsymbol{\rho}}\newcommand{\bisigma}{\boldsymbol{\sigma}}\newcommand{\bitau}{\boldsymbol{\tau}}\newcommand{\biupsilon}{\boldsymbol{\upsilon}}\newcommand{\biphi}{\boldsymbol{\phi}}\newcommand{\bichi}{\boldsymbol{\chi}}\newcommand{\bipsi}{\boldsymbol{\psi}}\newcommand{\biomega}{\boldsymbol{\omega}}{r^2} = 0.17$ and $\newcommand{\bialpha}{\boldsymbol{\alpha}}\newcommand{\bibeta}{\boldsymbol{\beta}}\newcommand{\bigamma}{\boldsymbol{\gamma}}\newcommand{\bidelta}{\boldsymbol{\delta}}\newcommand{\bivarepsilon}{\boldsymbol{\varepsilon}}\newcommand{\bizeta}{\boldsymbol{\zeta}}\newcommand{\bieta}{\boldsymbol{\eta}}\newcommand{\bitheta}{\boldsymbol{\theta}}\newcommand{\biiota}{\boldsymbol{\iota}}\newcommand{\bikappa}{\boldsymbol{\kappa}}\newcommand{\bilambda}{\boldsymbol{\lambda}}\newcommand{\bimu}{\boldsymbol{\mu}}\newcommand{\binu}{\boldsymbol{\nu}}\newcommand{\bixi}{\boldsymbol{\xi}}\newcommand{\biomicron}{\boldsymbol{\micron}}\newcommand{\bipi}{\boldsymbol{\pi}}\newcommand{\birho}{\boldsymbol{\rho}}\newcommand{\bisigma}{\boldsymbol{\sigma}}\newcommand{\bitau}{\boldsymbol{\tau}}\newcommand{\biupsilon}{\boldsymbol{\upsilon}}\newcommand{\biphi}{\boldsymbol{\phi}}\newcommand{\bichi}{\boldsymbol{\chi}}\newcommand{\bipsi}{\boldsymbol{\psi}}\newcommand{\biomega}{\boldsymbol{\omega}}{r^2} = 0.25$ for grader 1 and 2, respectively). The κ statistics showed a slight agreement between subjective and objective classification when Meiboscore limits criteria was used ($\newcommand{\bialpha}{\boldsymbol{\alpha}}\newcommand{\bibeta}{\boldsymbol{\beta}}\newcommand{\bigamma}{\boldsymbol{\gamma}}\newcommand{\bidelta}{\boldsymbol{\delta}}\newcommand{\bivarepsilon}{\boldsymbol{\varepsilon}}\newcommand{\bizeta}{\boldsymbol{\zeta}}\newcommand{\bieta}{\boldsymbol{\eta}}\newcommand{\bitheta}{\boldsymbol{\theta}}\newcommand{\biiota}{\boldsymbol{\iota}}\newcommand{\bikappa}{\boldsymbol{\kappa}}\newcommand{\bilambda}{\boldsymbol{\lambda}}\newcommand{\bimu}{\boldsymbol{\mu}}\newcommand{\binu}{\boldsymbol{\nu}}\newcommand{\bixi}{\boldsymbol{\xi}}\newcommand{\biomicron}{\boldsymbol{\micron}}\newcommand{\bipi}{\boldsymbol{\pi}}\newcommand{\birho}{\boldsymbol{\rho}}\newcommand{\bisigma}{\boldsymbol{\sigma}}\newcommand{\bitau}{\boldsymbol{\tau}}\newcommand{\biupsilon}{\boldsymbol{\upsilon}}\newcommand{\biphi}{\boldsymbol{\phi}}\newcommand{\bichi}{\boldsymbol{\chi}}\newcommand{\bipsi}{\boldsymbol{\psi}}\newcommand{\biomega}{\boldsymbol{\omega}}\kappa = 0.151,P \lt 0.001$ and $\newcommand{\bialpha}{\boldsymbol{\alpha}}\newcommand{\bibeta}{\boldsymbol{\beta}}\newcommand{\bigamma}{\boldsymbol{\gamma}}\newcommand{\bidelta}{\boldsymbol{\delta}}\newcommand{\bivarepsilon}{\boldsymbol{\varepsilon}}\newcommand{\bizeta}{\boldsymbol{\zeta}}\newcommand{\bieta}{\boldsymbol{\eta}}\newcommand{\bitheta}{\boldsymbol{\theta}}\newcommand{\biiota}{\boldsymbol{\iota}}\newcommand{\bikappa}{\boldsymbol{\kappa}}\newcommand{\bilambda}{\boldsymbol{\lambda}}\newcommand{\bimu}{\boldsymbol{\mu}}\newcommand{\binu}{\boldsymbol{\nu}}\newcommand{\bixi}{\boldsymbol{\xi}}\newcommand{\biomicron}{\boldsymbol{\micron}}\newcommand{\bipi}{\boldsymbol{\pi}}\newcommand{\birho}{\boldsymbol{\rho}}\newcommand{\bisigma}{\boldsymbol{\sigma}}\newcommand{\bitau}{\boldsymbol{\tau}}\newcommand{\biupsilon}{\boldsymbol{\upsilon}}\newcommand{\biphi}{\boldsymbol{\phi}}\newcommand{\bichi}{\boldsymbol{\chi}}\newcommand{\bipsi}{\boldsymbol{\psi}}\newcommand{\biomega}{\boldsymbol{\omega}}\kappa = 0.212,\,P \lt 0.001$ for graders 1 and 2, respectively). The limits established for Meiboscore did not consider the true distribution of the percentage of DOA and were set arbitrarily. Therefore, it becomes necessary to redefine the limits for the classification when performing automatic assessment of meibography. For this purpose, the percentage of DOA has been clustered in four classes so their intraclass variance is minimal using Otsu's classification algorithm.^35^ The number of classes was chosen due to legacy issues according with the number of grades of the conventional grading scales. The new classification resulted in the following intervals: Grade 0 – 0 ≤ DOA < 16%, Grade 1 – 16% ≤ DOA ≤ 32%, Grade 2 – 32% < DOA ≤ 59%, and Grade 3 – 59% < DOA.

For Grades 0, 1, 2, and 3, the mean ± 1 SD values of the objectively estimated percentage of DOA were 8.82 ± 4.70, 22.79 ± 4.11, 40.96 ± 7.01, and 69.50 ± 2.12, respectively. One clear observation is that that those results are now associated with lower standard deviations than those encountered when using Meiboscore.

**Figure 7:**
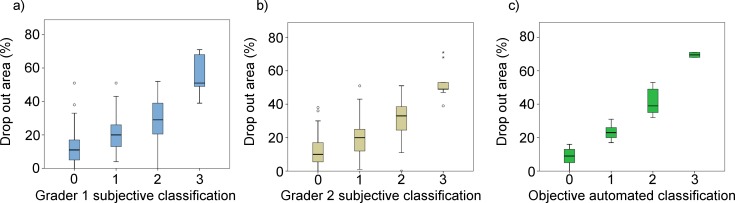
Box plots of the dropout area for the subjective classification according to graders 1 and 2 criteria (a) and (b), respectively, and the objective classification (c).

Figure 7 shows the box plots of the DOA variable for the four groups for both classifications (e.g., the subjective classification for both graders (Figs. 7a, 7b) and the objective classification (Fig. 7c). Although the difference between each pair of groups is statistically different using subjective and objective classifications (analysis of variance [ANOVA] with Tukey post hoc test, *P* < 0.010 assuming *P* < 0.05 was significant), with the automatic classification, the groups are more dissociated with less overlapping between them.

The Table shows the mean values and standard deviations for the extracted parameters arranged according to this new objective classification. As expected, the number of glands, and length and width of the glands are inversely proportional to the DOA. Length and number of glands clearly decrease when the DOA increases and groups are statistically significantly different as assessed by the Kruskal-Wallis test (*P* = 0.046 and *P* = 0.018, for number of glands and length respectively). Even that the difference in width values was lower, the Kruskal-Wallis test also revealed statistically significant differences (*P* = 0.015).

**Table:** Mean Values and Standard Deviations (in parentheses) for the Extracted Parameters According to the New Proposed Classification

Group	*N*	DOA, %	Number of Glands	Irregularity	Relative Length, %	Length, mm	Width, mm
0	68	8.8 (4.7)	26 (6)	21.92 (44.48)	70 (15)	2.9 (0.5)	0.4 (0.1)
1	53	22.8 (4.1)	25 (6)	22.22 (33.44)	61 (15)	2.6 (0.5)	0.4 (0.1)
2	26	41.0 (7.0)	19 (6)	8.59 (20.77)	45 (11)	1.8 (0.4)	0.3 (0.1)
3	2	69.5 (2.1)	14 (2)	0 (0)	26 (9)	1.3 (0.3)	0.2 (0.1)

### Gland Irregularity

**Figure 8:**
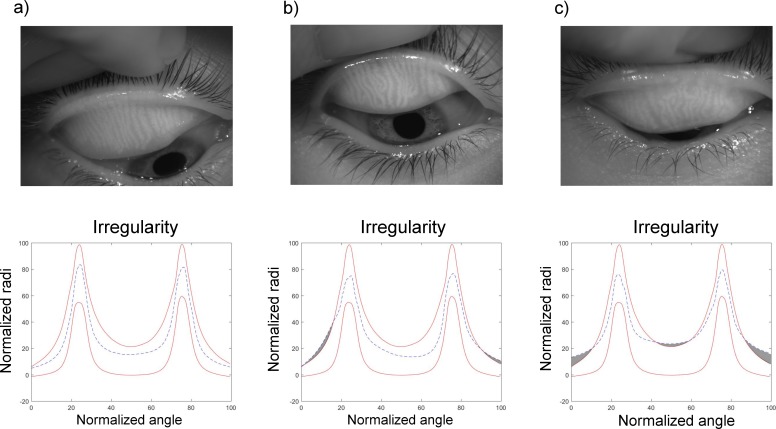
Example of the irregularity plot for three eyes with different amount of irregularity: low (a), medium (b), and high (c). The irregularity is given as the area enclosed between the blue dashed line out of the limits and the red lines, indicated as a gray shaded area.

Irregularity measurement is a novel approach that allows quantifying glands irregularity. Figure 8 shows three eyelids with different amount of gland irregularity, represented in the plots by the gray shaded area. As indicated in the Table, gland irregularity has large variability within each group, being higher for the group corresponding to smaller DOA and decreasing as the DOA increases. Not only the variability, but also the amount of irregularity is higher for those subjects exhibiting less glandular tissue loss.

## Discussion

This study describes a fully automated algorithm for analyzing meibography images that enables objective and quantitative assessment of MG. Except for improperly acquired images, the algorithm was successful, in terms of implementation (automatically identifying the ROI and isolating the glands), in its entire procedure in 98% of cases. For the remaining 2% (three images) where the algorithm failed, automated analysis of glands could be performed after manually selecting ROI.

Reliability of different grading scales, where the images are assessed subjectively, has shown to be modest.^20^ Some studies have used specific software, such as ImageJ (Wayne Rasband, National Institutes of Health, Bethesda, MD) that allows a semiautomatic assessment, where the area of MG loss is compared to the total area of the tarsal conjunctiva.^15^,^23^,^24^,^36^ However, the user still must delineate the area of the glands manually with the subsequent variability in gland assessment and increase in diagnostic time. Using an objective grading of the MG loss with a software, such as ImageJ, has shown to improve intra- and interobserver agreement.^22^ Image analysis techniques have been shown to improve the repeatability and accuracy of other subjective grading scales commonly used to assess different parameters of the ocular surface.^37^

To date, only few fully automated algorithms to analyze MG have been suggested. Koh et al.^38^ proposed the first algorithm to analyze meibography images. They were extracting morphologic features, such as gland length and spaces between glands, and combining them together with a linear classifier to differentiate between images of healthy and unhealthy individuals. However, this method was not fully automatic as the tarsal conjunctiva was selected manually. Later, Celik et al.^28^ proposed a new approach based on Gabor wavelets filtering with no user input needed. Likewise, in the previous study, glands and interglands length and width were used together with a support vector machine (SVM) to differentiate, in that case, between three levels of disease: healthy, intermediate, and unhealthy. This automatic classification was compared to the classification made by a clinician that was considered as the ground-truth; however, no details were reported on how the clinicians were classifying the images. Even though there were certain improvements with respect to the previous work, the ROI was not adapted to each image. Instead, it seemed to be a standardized elliptical area for all images. The details about how that ellipse was centered and positioned in the image are missing. Also, the use of SVM with small samples should be interpreted with caution.

The second automated algorithm was proposed by Arita et al.,^27^ where image enhancement techniques were used to isolate the glands and compute the ratio between the area occupied by glands and the total area of analysis to estimate the DOA. This ratio was calculated for each of the four Meiboscore groups^18^ finding statistically significant differences between any combination of two groups. However, using Meiboscore classification, the difference between the group with Meiboscore 0 and 1 was small. In our study, the same problem was faced when dividing the cohort according to the Meiboscore limits. According to Meiboscore criterion, one can only classify a subject as grade 0 if there is 0% of DOA. If there is, for example, 1% of MG dropout, the automated algorithm would classify this subject as Meiboscore 1, whereas a clinician would most likely classify it as Meiboscore 0, as this small dropout would be difficult to notice. To overcome this, new limits to classify the grade of MG loss have been proposed. As evidenced by the results, this new classification is optimized to better separate the groups by minimizing their intraclass variation.

Recently, Koprowski et al.^25^,^26^ suggested two different approaches to automatically analyze meibography images. In both, they were comparing the area with detected glands to the total area of analysis and the capability of this measure in dry eye diagnosis, dividing the cohort in three categories: healthy, at-risk, and unhealthy subjects. These results were compared to a clinician judgment while the clinician criteria used for dry eye evaluation were not reported.

The proposed algorithm provides a three-class classification and it is able to differentiate between gland, intergland and DOA. It also excludes from the analysis the reflections on the tarsal conjunctiva. The algorithm provides an estimate for the DOA and also other morphologic parameters of MG that, combined with the DOA, could add diagnostic value to the assessment of the images and to the follow up of MGD progression.^39^ In fact, some studies have reported that a progressive dysfunction of MG causes morphologic glandular changes^40^,^41^ and also a correlation of MG length and width to some tear film parameters.^42^

To the best of our knowledge, this is the first time that an automated method quantifies the irregularity of the glands. Gland irregularity has been claimed to be a valuable feature especially for follow-up of the progression of MGD.^39^–^41^ We found that there is a large variability in the irregularity measurements, especially in Groups 0 and 1. This is not surprising as the less glands and shorter the glands are, the less chance they have to be irregular. The results suggested that there might be changes in the number of glands, gland length, width, and irregularity, related to the degree of MGD. However, further investigation is needed to evaluate the diagnostic capability of the suggested parameters and how they relate to other ocular surface indicators to ascertain whether they could add value to the MG assessment and enhance the MGD diagnosis and follow-up.

The study has some limitations. First, the current version of the algorithm is instrument-specific (Keratograph 5M), because the majority of parameters were selected empirically for the given image size. Nevertheless, for other instruments with different image resolution and other potential differences, such as level of illumination, the proposed algorithm can be adapted easily. Secondly, for this study, only the upper lid was considered. Since evaluation of both lids could have better diagnostic performance,^23^,^24^ future work will adapt the algorithm for lower lid analysis. Thirdly, one limitation, inherent to the meibography in general, is that each time the eyelid is everted, the exposed tarsal conjunctiva is different having different amount of area and shape. This implies that there could be slight differences in the calculated parameters, and also that the longitudinal assessment in time of MG changes is subject to the way clinician is everting the eyelid. This could be overcome by automatically recognizing and matching the images taken in different sessions so local changes could be tracked, contributing to more accurate monitoring of MGD. Finally, even though the sample population was considered to be representative of the general population in terms of age, the percentage of contact lens users in this study was higher than that reported in developed countries.^43^ It is debatable whether contact lens wear influences MG dropout and MG morphology.^44^ If so, the data in this study would be skewed towards higher levels of dropout and irregularity. To avoid this potential bias, the number of contact lens users should be controlled. However, more long-term studies are needed to ascertain whether contact lenses have long-term effects on MG. Automated and objective methods may represent an improvement in the assessment of MG changes over time, particularly in cases of the follow-up measurements that must be related to their predecessors.

## Conclusions

A new automated methodology to analyze infrared meibography images has been proposed. It performs a morphometric analysis of the MG and an estimation of the DOA. Also, new limits for grading DOA have been proposed. The proposed methodology overcomes the limits of subjective assessment of MG enabling noninvasive, automatic, and precise evaluation of DOA and their morphologic characteristics. These analyses could be useful in MG characterization and assessment, reducing the variability and time associated with subjective judgment.
